# A Combined MPI-CUDA Parallel Solution of Linear and Nonlinear Poisson-Boltzmann Equation

**DOI:** 10.1155/2014/560987

**Published:** 2014-06-12

**Authors:** José Colmenares, Antonella Galizia, Jesús Ortiz, Andrea Clematis, Walter Rocchia

**Affiliations:** ^1^Drug Discovery and Development, Italian Institute of Technology, 16163 Genova, Italy; ^2^Department of Geosciences, University of Padova, 35131 Padova, Italy; ^3^IMATI, CNR, 16149 Genova, Italy; ^4^Department of Advanced Robotics, Italian Institute of Technology, 16163 Genova, Italy

## Abstract

The Poisson-Boltzmann equation models the electrostatic potential generated by fixed charges on a polarizable solute immersed in an ionic solution. This approach is often used in computational structural biology to estimate the electrostatic energetic component of the assembly of molecular biological systems. In the last decades, the amount of data concerning proteins and other biological macromolecules has remarkably increased. To fruitfully exploit these data, a huge computational power is needed as well as software tools capable of exploiting it. It is therefore necessary to move towards high performance computing and to develop proper parallel implementations of already existing and of novel algorithms. Nowadays, workstations can provide an amazing computational power: up to 10 TFLOPS on a single machine equipped with multiple CPUs and accelerators such as Intel Xeon Phi or GPU devices. The actual obstacle to the full exploitation of modern heterogeneous resources is efficient parallel coding and porting of software on such architectures. In this paper, we propose the implementation of a full Poisson-Boltzmann solver based on a finite-difference scheme using different and combined parallel schemes and in particular a mixed MPI-CUDA implementation. Results show great speedups when using the two schemes, achieving an 18.9x speedup using three GPUs.

## 1. Introduction

The Poisson-Boltzmann equation (PBE) describes the electrostatic behavior of a polarizable solute containing fixed charges immersed in an ionic and polarizable solution. It is a popular and effective model adopted in the computational structural biology and biophysics communities, where the estimate of the electrostatic energy of molecular systems is used, for instance, to study stability, binding affinity, desolvation penalty, acid constants, and so forth [1, 2]. The last decades witnessed a remarkable increase of the available structural data concerning biomolecules. Concurrently, web servers and databases started accumulating annotations and derived/simulated quantities related to the original experimental structures. A similar thing has also been done as far as the electrostatic potential generated by entries of the Protein Data Bank is concerned [3, 4]. Nowadays, the amount of data is still increasing and it is complemented by the availability of molecular dynamics simulation outcomes, resulting in a further explosion of the number of structures. Moreover, researchers started using the same approaches once devised for small sized proteins to more complex and larger scale systems, such as multimeric receptors in cell membrane, virus capsids, and ribosomes [5]. This is instrumental to the creation of databases of paired experimental and simulated data necessary to a better comprehension of biological and medical data.

The need to analyze larger number of bigger and more complex systems translates into the need for faster algorithms. The parallelization of algorithms has become mandatory to take advantage of modern computational architectures. In recent years, the evolution and growth of the techniques and platforms commonly used for high performance computing (HPC) have been truly astonishing [6]. Multicore processors are now ubiquitous; the famous Moore Law [7], stating that the number of transistors on integrated circuits doubles approximately every year, readjusted every two years (http://news.cnet.com/2100-1001-984051.html), can still be considered valid, but with a major change: every new generation of CPUs is more powerful than the previous one mostly because it provides more cores. Furthermore, the last generation CPUs can also access powerful specialized hardware, as general-purpose graphics processing units (GPGPUs, shortly, GPUs) and field-programmable gate arrays (FPGAs) [8]. These emerging heterogeneous HPC architectures provide significant computational power, in the order of TFLOPS. However, this raw potential can become practically available only through a massive exploitation of parallelism, which requires a tailored approach for each architecture. For this aim, several different paradigms and libraries have been designed [9].

In this context, we present an implementation of a full PBE solver based on a finite-difference (FD) scheme using different parallelization schemes and in particular a combined MPI-CUDA implementation. We follow the approach of the DelPhi PBE solver [10, 11], which exploits the checkerboard structure of the finite difference discretization of the Laplace differential operator and adopts a successive overrelaxation (SOR) scheme to converge to the solution. Our implementation exploits two levels of parallelism; thus it makes it possible to exploit multicore CPUs and clusters of CPUs as well as (multi-)GPUs and clusters of GPUs. The paper is organized as follows. First, a basic description of the PBE and a sequential solution scheme are given. Then, existing paradigms and libraries for parallelization on heterogeneous architectures are introduced. Sections 4, 5, and 6 describe the parallel implementations proposed to solve PBE using GPUs, clusters of CPUs using MPI, and the combined use of MPI and CUDA.  Section 7 details the combined use of MPI and OpenMP.  Section 8 presents and discusses the experimental results achieved, while Section 9 concludes the paper.

## 2. Sequential Solution of the Poisson-Boltzmann Equation

The PBE combines the continuum electrostatics description of fixed charges in a dielectric medium with the Boltzmann prescription for mobile ions in aqueous solvent at the thermal equilibrium with a reservoir [12]. In its linearized form, which is valid for low ionic concentrations, the PBE reads

(1) $$\begin{array}{c} \;\nabla \cdot \left[\epsilon \left(x\right)\nabla \Phi \left(x\right)\right]+\frac{\rho^{\text{fixed}}}{\epsilon_{0}}=\frac{\epsilon_{\text{solv}}}{\lambda^{2}}\Phi \left(x\right), \end{array}$$

where Φ is the electrostatic potential, *ϵ*(**x**) the space-varying relative dielectric constant, *ϵ*_solv_ that of solvent, *ϵ*_0_ the permittivity of vacuum, *ρ* ^fixed^ the fixed charge density on the solute, and *λ* the Debye length of the ionic solution, a quantity describing the electrostatic screening induced by the ionic cloud in the solution. The right hand side of (1) is present only if **x** is located in the ionic solution. The sequential implementation described here follows the approach described in [10]. The PBE discretized on a uniform grid takes the following form:

(2) $$\begin{array}{c} \left[\overset{6}{\underset{i=1}{\sum }}\epsilon_{i}+\epsilon_{\text{solv}}{\left(\frac{h}{\lambda }\right)}^{2}\right]\Phi_{j}-\overset{6}{\underset{i=1}{\sum }}\epsilon_{i}\Phi_{i}-\frac{q_{j}}{\epsilon_{0}h}=0, \end{array}$$

where Φ_*j*_ refers to the electrostatic potential at the node *j*, where a net charge *q*_*j*_ is mapped. The term containing *λ* is present only if the node *j* belongs to the solvent and *ϵ*_*i*_ is the relative dielectric constant at one of the midpoints between the node *j* and its six nearest neighbors on the grid; *h* is the grid spacing. This discretized relationship leads to a linear system of equations *A*Φ = *b* where a suitable mapping converting three-dimensional to one-dimensional indexes has to be adopted. The matrix *A* can then be decomposed into *A* = *D* + *L* + *U*, where *D* is the diagonal of *A* and *U* and *L* are the strict upper and lower triangular parts of *A*, respectively. According to the successive overrelaxation method, the iterative equation is given by

(3) $$\begin{array}{c} \Phi^{\left(n+1\right)}={\left(D+\omega L\right)}^{-1}\left\{\omega b-\left[\omega U+\left(\omega -1\right)D\right]\Phi^{\left(n\right)}\right\}, \end{array}$$

where *ω* is the overrelaxation factor and bracketed superscripts indicate iteration number. The term (*D*+*ωL*)^−1^ can be calculated using forward substitution since *D* + *ωL* is a lower triangular matrix implying that the iterative scheme must be consistent with the previously described mapping, which makes parallelization difficult. The iteration stencil becomes

(4) $$\begin{array}{c} \Phi_{j}^{\left(n+1\right)}=\omega \left(\frac{\sum_{i=1}^{6}\epsilon_{i}\Phi_{i}^{\left(n\right)}+\left(q_{j}/\epsilon_{0}h\right)}{\sum_{i=1}^{6}\epsilon_{i}+\epsilon_{\text{solv}}{\left(h/\lambda \right)}^{2}}\right)+\left(1-\omega \right)\Phi_{j}^{\left(n\right)}. \end{array}$$

The best overrelaxation factor can be obtained from the highest eigenvalue of the iteration matrix [13], which in turn can be calculated using the connected-moments expansion [10]. This stencil was first used in [2] and a revision of its uses (at the time of writing) can be found in [14]. Later, the stencil has been parallelized using MPI in [15] and using CUDA but with different kernels in [16, 17].

In order to obtain a well-defined solution, suitable boundary conditions must be ensured; the interested reader can find some details on different available alternatives in the work of Rocchia, which focuses on biological applications [18].

### 2.1. Solving the Nonlinear PBE

To solve the nonlinear PBE, the nonlinearity is treated as a perturbation to the linear counterpart:

(5) ∇·[ϵ(x)∇Φ(x)]−ϵsolvκ2(x)Φ(x)   =−ρfixed(x)ϵ0+ϵsolvκ2(x)[sinh⁡(Φ)−Φ].

This allows making a minor adaptation of the linear solver by gradually introducing the nonlinearity. The stencil for the nonlinear solver thus reads

(6) $$\begin{array}{c} \Phi_{j}^{\left(n+1\right)}=\omega \left(\frac{\sum_{i=1}^{6}\epsilon_{i}\Phi_{i}^{\left(n\right)}+\left(q_{j}/\epsilon_{0}h\right)+\xi_{j}}{\sum_{i=1}^{6}\epsilon_{i}+\epsilon_{\text{solv}}{\left(h\kappa \right)}^{2}}\right)+\left(1-\omega \right)\Phi_{j}^{\left(n\right)}, \end{array}$$

where *ξ* accounts for the nonlinearity. This procedure is currently employed in the sequential DelPhi software and it is better described in [11].

### 2.2. Exploiting the Structure of the System

The following observations help significantly to improve the efficiency of the algorithm. First, if the number of grid points in the first two dimensions is odd, the discretized FD scheme is endowed with the so-called checkerboard structure. All even grid points depend only on their neighboring grid points, which are odd, and* vice versa*. This allows iterating alternately on grid points of different parity until convergence. Due to this property, one can break the dependence imposed by formula (3) and apply the parallelism inside each of the even/odd steps. Second, it is worth pointing out that on most grid points no charges are mapped and also are located in a uniform dielectric region, where *ϵ*_*i*_ is constant. In most of the cases, indeed, *ϵ* varies only around the molecular surface. Due to these observations, the stencil can be simplified as follows:

(7) $$\begin{array}{c} \Phi_{j}=\frac{\sum_{i=1}^{6}\Phi_{i}}{6+\kappa_{j}^{2}}, \end{array}$$

where

(8) $$\begin{array}{c} \kappa_{j}=\{\begin{array}{cc} \left(\frac{h}{\lambda }\right) & \text{if}\;j\;\text{is}\;\text{inside}\;\text{the}\;\text{ionic}\;\text{solution},\\ 0 & \text{otherwise}, \end{array} \end{array}$$

allowing a faster parallelization. After each run of this uniform stencil, corrections have to be made at the points where charges are present and where *ϵ*_*i*_ changes. This solution is therefore faster than using the full nonuniform stencil on the whole grid.

#### 2.2.1. Contiguous Memory Mapping

Instead of making the numerical computations and moving the memory access along a three-dimensional parallelepiped and updating the odd and even points, the solution was calculated using two 1D pointers: one for the even and one for the odd grid points, Φ_*e*_ and Φ_*o*_, respectively. Every grid point *p*_*o*_(*x*_*o*_, *y*_*o*_, *z*_*o*_) is mapped into an odd *p*_odd_ or even *p*_even_ pointer according to the rule

**Figure 1:**
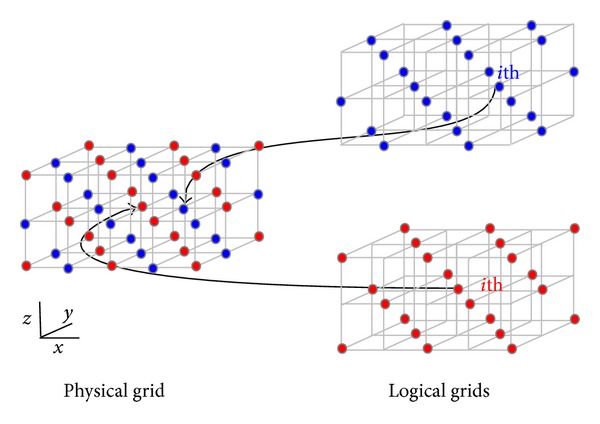
Checkerboard structure used to build the continuous memory mapping.

(9) peven=xo+nxyo+nxnyzo2,podd=xo+nxyo+nxnyzo−12

so that the update of each pointer depends only on the one with opposite parity. The offset of the indexing of the neighboring points in this case can be seen in Table 1. In Figure 1, we show a 3D graphical representation of the checkerboard structure and its relationship with the arrays used for the continuous memory mapping.

### 2.3. Sequential Algorithm

Due to the corrections that have to be made after the uniform stencil is applied, namely, on the regions where the dielectric constant is not uniform and where charges are present, a preprocessing stage is needed to identify the pointers corresponding to the grid points located in these regions. These steps are as follows.

- *Determine inside/outside*: Determine which grid points are on the solute or in the solvent; this involves the calculation of the molecular surface of the solute (see [19] for a summary of the different possibilities). If there is salt in the solution, we also calculate the *κ* factor.
- *Find dielectric boundaries and prepare the boundaries correction*: Look for the midpoints in which *ϵ*_*i*_ varies and calculate the correction to be applied after the stencil operation
- *Set boundary conditions*: Set up the boundary conditions to be used; see [18] for a description of the possibilities.
- *Prepare charges correction*: Calculate the correction to be applied to the grid points where charges have been assigned.

After that, the main iteration then applies the uniform Laplace stencil to the grid points of one parity, and, afterwards, it corrects it where needed. Then, the opposite parity points are updated, with the corresponding correction. The convergence of the iterative scheme is evaluated using the maximum difference of the potential on the grid every 10 iterations. The steps of the main loop are the following.

1. *Save dielectric boundaries*: Save the state of the dielectric boundary points considering a temporary vector for convergence test at the end.
2. *Run Poisson or Poisson-Boltzmann*: This is the main calculation block that implements the stencil given by (7) and is executed on every grid point.
3. *Adjust dielectric boundaries*: Update the potential value of the grid points located at the dielectric boundary; this is done at the end of each iteration.
4. *Add charges*: Add the charge terms to the grid points that were predefined as charged.
5. *Calculate potential difference at the dielectric boundary*: Calculate the absolute differences between the current potential values at the dielectric boundary with the one saved previously on a temporary data structure. This is done since the boundary is the region where the convergence is expected to be slower.
6. *Check convergence*: The maximum absolute difference between the potential at two subsequent iterations is compared to the threshold to test the convergence and to decide whether to stop the iterative procedure.

In the rest of the paper, for each parallel implementation of the full Poisson-Boltzmann solver, we refer to this list to explain the adopted approach.

## 3. Heterogeneous Computing Systems and Parallel Programming Libraries

As previously highlighted, in a modern complex computing system, the computational cores, memory banks, and communication bandwidth can be extremely heterogeneous. To get the expected level of performance, it is mandatory to manage effectively such intrinsic architectural complexity. For this reason, the actual barrier posed by heterogeneous HPC resources is the difficulty in the development and/or the performance efficient porting of software on such complex architectures [9, 20]. The traditional HPC solutions offer widely used programming models and tools, since such parallel computing systems have now achieved certain maturity thanks to high-level libraries, for example, ScaLAPACK [21], or runtime libraries as MPI [22], while new heterogeneous architectures require an effort in the development of customized solutions. In fact, the efficient exploitation of hierarchical and heterogeneous architectures requires an increased effort in software development and presents challenges also in terms of the scalability of applications.

The view of heterogeneous computational systems corresponds to different types of parallel cooperation among parallel processes: distributed memory for cooperation among nodes, shared memory for core cooperation, and SIMD (single instruction multiple data) parallelism inside CPUs and accelerators (GPUs). The challenge is the development of parallel applications able to exploit in an effective way these different levels of parallelism; in particular, in this work we use MPI, OpenMP, and CUDA.

MPI (message passing interface) is a language-independent communication library used to program parallel computers. It supports explicit communication among processes that constitute a parallel program running on a distributed memory system. Communications can be both point-to-point and collective. MPI goals are high performance, scalability, and portability. MPI implementation can be smart enough to realize that it runs on a shared memory environment and consequently to optimize its behavior accordingly. Designing programs that adopt the MPI model (contrary to explicit shared memory models) may have advantages over nonuniform memory access (NUMA) architectures since MPI encourages memory locality.

The Open specifications for Multi-Processing (OpenMP) define a set of compiler directives, library routines, and environment variables that can be used to specify shared memory parallelism in Fortran and C/C++ programs. It is based on compiler directives and it offers a simple and elegant paradigm for supporting core-level and CPU-level parallelism. Transition from sequential to parallel is extremely easy and smooth, since it supports a unified code for both sequential and parallel applications: OpenMP constructs are treated as comments when sequential compilers are used. One drawback of OpenMP is that it currently runs efficiently only on shared-memory multiprocessor platforms; thus the main option for clusters remains MPI. It is to underline that with the proper policy OpenMP could also fix the NUMA issues [23].

The compute unified device architecture (CUDA) is a parallel computing platform and programming model created by NVIDIA that gives developers access to the instruction set and memory of the parallel computational elements in NVIDIA GPUs. CUDA is accessible to software developers through CUDA-accelerated libraries, compiler directives, and extensions to programming languages such as C/C++, Fortran, and other interfaces. CUDA acts at a lower architectural level compared with the previous tools, and thus it requires higher programming skills.

## 4. CUDA Implementation

The CUDA implementation was developed to speed up the computation of (7) exploiting GPU. GPUs are highly parallel programmable microprocessors, originally born to support graphics elaboration; they are used in combination with CPU as a coprocessor to speed up numerically intensive parts of code by the means of a massive fine grained parallelism. The parts of the code that exhibit a rich amount of data parallelism are performed on the GPU in a SIMD mode; data have to be transferred to the GPU memory; this transfer represents the actual bottleneck of the computation, and programs (kernels) directly targeting GPUs have to be written. Using CUDA, a kernel is executed on threads organized in blocks; each thread is responsible for a portion of data, and each block of threads shares a local memory (called shared).

In the CUDA implementation of the solver, we followed the algorithm described in [16, 24], where CUDA was used to parallelize the main iteration, while the preprocessing was calculated in the sequential part of the code. Memory transfers in the main loop occur every several iterations (typically 10) to test the convergence. We used the GPU shared memory that is faster than the global memory. The limiting factor on the use of this optimized memory is mainly the size, that is, 16 KB in architectures before Fermi GPU or 48 KB on Fermi architectures and onwards; however, it was sufficient for our aim.

In [25], authors explore stencil computations to optimize the Jacobi method for solving Laplace's differential equation using different programming models and in particular CUDA. One of the solutions proposed exploiting CUDA is quite close to ours at least in terms of thread organization and in the use of shared memory. However, authors improve the level of the discussion deeply exploring further optimization policies, just to name a few: the use of internal register (instead of the shared memory) thus avoiding synchronization barriers among threads in the same block and the application of tiling strategies. We moved instead to the combined use with the MPI implementation.

**Figure 2:**
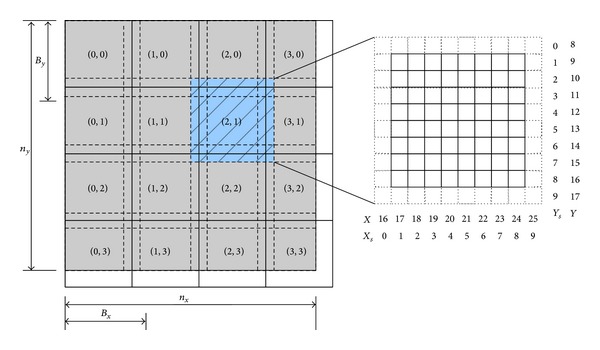
* Tartan* distribution of the blocks of threads in CUDA.

The points of the grid can be accessed through their coordinates: *X*, *Y*, and *Z* are the global coordinates on the whole volume, that is, the same used in the sequential algorithm. Each thread is associated with a set of points with fixed *X* and *Y* coordinates, moving along the *Z* coordinate and skipping the points of opposite parity. A bidimensional distribution of threads and blocks has been implemented; in Figure 2, we can see this distribution for each value of *Z* coordinate. The points of the grid inside the continuous lines are actually updated, while the dotted lines mark the neighborhood points required in the computation. The size of the blocks is *Bx* and *By*; in our implementation, a block size of 16 × 16 was empirically found to provide the best results. *X*_*s*_ and *Y*_*s*_ correspond to the local coordinates of the grid point inside the blocks; these are used during the CUDA computation by the thread to relate to the actual grid points. The coordinates of the points are calculated in the following way, as for the local ones *X*_*s*_ and *Y*_*s*_,

(10) Xs=threadIdx.xYs=threadIdx.y,

while the global *X* and *Y*

(11) $$\begin{array}{c} X=threadIdx.x+blockIdx.x\cdot \left(blockDim.x-2\right)\\ Y=threadIdx.y+blockIdx.y\cdot \left(blockDim.y-2\right), \end{array}$$

where (following the CUDA notation) *threadIdx* are the 2D coordinates of the thread inside the block, *blockIdx* are the 2D coordinates of the block inside the grid, and *blockDim* is the dimension of the block. From the three coordinates *X*, *Y*, and *Z*, one can derive the index in the linear buffer where the potential is stored, as shown in (12). This relationship is the same for even and odd points:

(12) $$\begin{array}{c} \text{index}=\left\lfloor \frac{X+Y\cdot n_{x}+Z\cdot n_{x}\cdot n_{y}}{2}\right\rfloor . \end{array}$$

Each thread, including those that are in the border area, copies the value of the grid point with opposite parity that has the same index to the shared memory. After that, all the threads of the same block synchronize to be sure that the shared memory is updated for all of them. Then, the interior points update their values according to the Laplace rule. The 1D coordinates of the six neighbors are obtained properly manipulating values presented in Table 1. While applying the Laplace stencil, the* Left*,* Right*,* Back,* and* Front* points are already in the shared memory, so we use the corresponding offset in the shared space. However,* Bottom* and* Top* points are missing, so we have to read them from the global memory using the offset in the global representation. Since we are iterating in the *Z* coordinate, we can reuse the* Top* information since it corresponds to the* Bottom* point of the next *Z* coordinate. So, in each iteration, we rewrite the* Bottom* point with the previous* Top* point and we read a new value with the indicated offset. In Algorithm 1, we schematize the approach we adopted.

As we can see in Figure 2 the grid (shaded in gray) could be smaller than the blocks of threads. This is because the blocks have the same dimension and it is not always possible to fit them into the grid dimension. The threads in charge of such data will be idle for a while.

The described CUDA implementation has been linked to the DelPhi software and is downloaded from http://www.electrostaticszone.eu.

## 5. MPI Implementation

The implementation described in this section was developed to enable the run of the numerical solver on distributed memory architectures such as cluster of (multicore) CPUs. This kind of architectures can be exploited using well-known SPMD (single program multiple data) programming model on distributed memory resources; the standard* de facto* in this context is MPI.

The approach adopted considers a data parallelism; that is, the global data set is subdivided in partial data sets elaborated in parallel. The volume storing input data was subdivided in smaller parallelepipeds; the number of the subdomains relies on the number of MPI processes spawned for the computation; in fact, each subdomain is assigned to a MPI parallel process that is in charge of its elaboration. The volume subdivision among the parallel processes was implemented using the parallel I/O functionalities provided by MPI (version 2 and onwards). The exploitation of this feature enabled the speedup of data distribution; in fact, we avoided the master-slave approach; that is, only one process (the master) accesses the data set and distributes data among the other processes (the slaves), that results in a more time consuming phase. Furthermore, the use of the MPI2 parallel I/O ensures optimized parallel accesses the data set thus reducing data contention.

In the checkerboard structure used to solve the Laplace equation, there is the need to consider for each point of the grid its 6 neighbors of opposite parity. Therefore, subdomains have to take into account overlapping areas to properly manage this requirement, and MPI data communications were introduced to exchange the neighbor points at the border of each subdomain. This marks a different approach from the one taken at [15], where authors used the direct remote memory access (DRMA) to manage the neighbor points.

The domain was divided along the most external dimension, that is, *Z*. Therefore, the dimensions of the subdomain elaborated by the MPI processes are parallelepipeds with the same *X* and *Y* dimensions but with a lower number of layers on *Z*. For each subdomain, also charges, dielectric boundaries, and the value of *κ* were assigned. Note that the communications involve even and odd grid points, so particular care is devoted to enforce the consistency of the parity of the points. In fact, since each subdomain acts as an independent solver, all subdomains assume that the first grid point is even. This has to be ensured during the subdivision of the domain. This problem was solved by dividing the domain so that each subdomain starts with an even grid point and a consequent management in the whole data set.

Defining *N*_*z*_ as the total number of levels/layers on the *z*-axis of the whole domain, *nproc* the number of MPI processes spawn, and *i* the process identifier, the algorithm employed to calculate the number of layers *n*_*z*_^*i*^ on the subdomain *i* is described in Algorithm 2.

To understand how the algorithm works, note that many conditions have to be imposed for the parity consistency and to properly manage the boundary requirements; for example, the first and the last *Z* levels on the whole volume have only one border to consider, while the other levels have to allocate two borders, one layer above and one below.

Once each subdomain is constructed, the solver acts in each subdomain almost as the sequential version would, and only minor modifications are needed. The boundary conditions on the faces perpendicular to the *x*- and *y*-axes are calculated as in the sequential case, and the boundary on faces perpendicular to *Z* requires the values that have to be exchanged exploiting MPI except for the first and last subdomains, where one of the faces actually corresponds to a boundary. A border is composed of 2 layers (one for each overlapping subdomain); thus, for each iteration, 4 layers have to be sent and 4 received. This is done after the update of the potential for each parity. Since the data transferred is needed right after the data communication occurs, only* blocking communications* were used.

## 6. Combining the MPI and CUDA Implementations

This implementation of the solver aims at exploiting the computing power of clusters of GPUs, that is, clusters, where nodes are equipped with one or more GPUs. This was investigated through a proper integration of the two independent implementations based on CUDA and MPI. The idea is to add a further level of parallelism to the previous implementations; that is, MPI is used to distribute the computation and CUDA as the main execution engine. This approach has been already adopted in the scientific community to speed up compute intensive tasks with successful results [26–28].

The integration of MPI and CUDA worked quite smoothly thanks to the experience matured with the previous parallel implementations, and we can affirm that it was not particularly intrusive. The original sequential part of the MPI implementation was replaced with the CUDA parallel kernel combining the related parallelization strategies. In particular, the data communications among MPI processes have to be carefully combined with CUDA data transfers from and to GPU memory to ensure the elaboration of the most up-to-date values and thus data consistency.

In more detail, the algorithm implements the MPI subdomain definition thus distributing data among MPI parallel processes. Each MPI process calls the GPU solver to elaborate its own subdomain. This means that data are transferred to the GPU and elaborated according to the CUDA implementation. As already outlined, MPI communications have to be consistent with data transfers to/from GPU memory. Since MPI blocking communication occurs at each iteration (4 layers have to be sent and 4 received), data transfer from/to GPU memory has to be performed at each iteration as well.

The use of nonblocking communications represents an interesting point to be considered. In fact, although the time spent on data transfer was small when using InfiniBand, thread synchronization was still needed because a blocking MPI communication model was used; therefore, nonblocking MPI could improve the speedup of the algorithm even further. In [29], a CUDA parallelization of the 3D finite difference is presented together with the use of MPI to enable the exploitation of multi-GPUs. In particular, wave equations, that are of great interest in seismic computing, are considered; of course, they pose different requirements to the geometry of the data set that lead to a different approach in the CUDA parallelization. MPI instead is used with a strategy similar to the one proposed in this section. However, authors obtain significant performance by overlapping data exchange among GPUs with kernel execution. In [30], mixed MPI-CUDA implementation is proposed along with the investigation of different strategies to improve the efficiency of incompressible flow computations. The data partition applied is quite close to ours. However, they also consider a strategy of overlapping computation between MPI and CUDA. This is obtained by modification of the kernels that have to be organized in such a way to enable (1) the overlap of the CUDA computation with the GPU data transfer/MPI communication and (2) the asynchronous execution of different kernels. The design of such a sophisticated algorithm pays in terms of performance achieved. Starting from these interesting experiences, we will investigate the use of nonblocking communications as a future direction.

The described strategy works with several GPUs per node, associating one MPI process for each GPU. With the CUDA toolkit 5, the GPUDirect feature has been introduced to achieve these goals easily and efficiently. Exploiting the GPUDirect, it is possible to directly send data from the GPU memory to a network adapter without staging through host memory; that is, MPI communications can involve data directly stored on GPUs. This is commonly known as CUDA-aware MPI. In the presented implementation, we did not have the possibility to test this feature and evaluate the performance gained in this way; we plan this step as a future investigation.

## 7. MPI versus OpenMP Implementations

In the case of one multicore node, that is, shared memory cores, it is interesting to compare the performance achievable using two different parallel libraries to exploit a shared memory architecture, as MPI and OpenMP. For this reason, we develop an OpenMP implementation of the solver.

The parallelization on multicore nodes using OpenMP is the easiest approach to implement. Pragma clauses were added at FD stencil, as it is by far the more computationally expensive part of the code. This can be considered equivalent to subdividing the volume containing the input dataset in cuboids, as done in the MPI parallelization and described in Section 5. Static scheduling was used, setting the chunk of each thread manually so that they are evenly distributed.

As in the CUDA, we combined this implementation with the one developed using MPI thus to exploit cluster of multicore CPUs; again, the effort spent to integrate the codes was actually affordable. The algorithm implements the MPI subdomain definition, thus distributing data among MPI parallel processes; on each data set, the OpenMP code is executed, while the MPI process manages data communications among the nonshared memory nodes. In that way, MPI controls the communication between nodes and OpenMP the parallelization in each node.

## 8. Experimental Results

We had the possibility to test the implementations described in this paper on several parallel resources corresponding to different architectures. The configuration of each resource can be seen in Table 2. Clusters 1 and 2 were used to test the MPI implementation and resource 3 was used to test the MPI-CUDA implementation. More information on the results on the GPU parallelization is discussed in [16, 24]. All tests used the same molecule with the same parameters: a fatty acid amide hydrolase molecule that, once ported to the cubic grid, consisted of 29880 charges on 297 × 297 × 297 grid points. A salt concentration of 0.15 mol/L and dipolar boundary conditions were used. In the following subsections, we discuss the different results obtained by adopting the implementations previously presented.

### 8.1. MPI Performance

**Figure 3:**
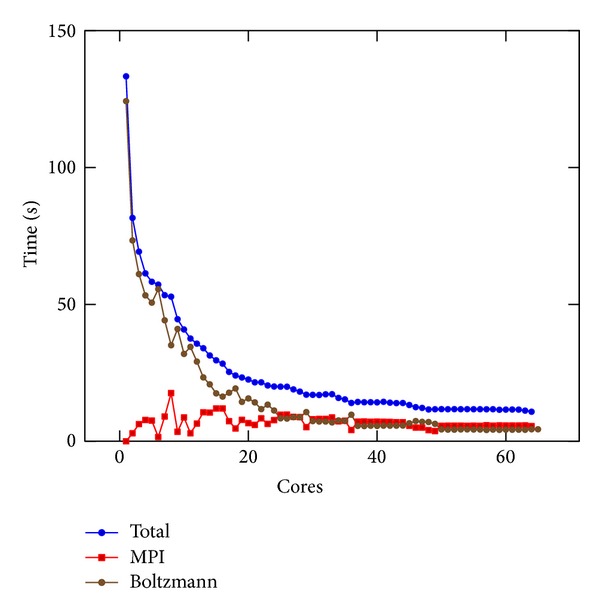
MPI results on Cluster 1. The whole execution time is depicted with a blue line; the time required for data communication is indicated as MPI and depicted with a red line; the stencil part of the solver is indicated as Boltzmann and depicted with a black line.

**Figure 4:**
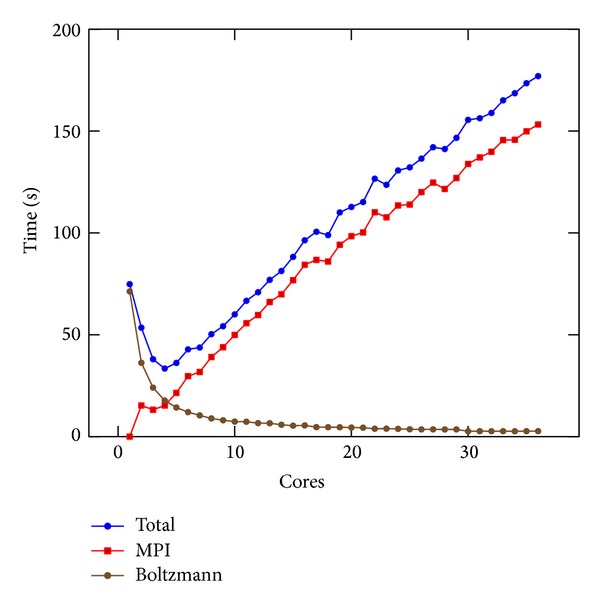
MPI results on Cluster 2. The whole execution time is depicted with a blue line; the time required for data communication is indicated as MPI and depicted with a red line; the stencil part of the solver is indicated as Boltzmann and depicted with a black line. When this figure is compared with Figure 3, InfiniBand proved to be crucial. Its absence meant that more time was spent on data communication rather than doing calculations.

In Figures 3 and 4 we present the results of Section 5 obtained on Cluster 1 and Cluster 2, respectively. Both refer to the linear PBE. In order to appreciate the impact of the different parts of the algorithm, we differentiate between the whole execution time, depicted with a blue line, the time required for data distribution and communication, indicated as MPI and depicted with a red line, and the time used on the stencil, indicated as Boltzmann and depicted with a brown line. The calculation of the Boltzmann stencil was the most demanding one in terms of execution time; the time spent on communications instead depends on the resource. In fact, it is possible to notice that, on Cluster 2, the absence of InfiniBand was heavily affecting computation since more time was spent in data transfer than iterating at the Boltzmann stencil, by far the most computationally expensive part of the algorithm. To make a fair comparison of the results obtained on the different resources, the time spent in MPI communications was not more reported.

**Figure 5:**
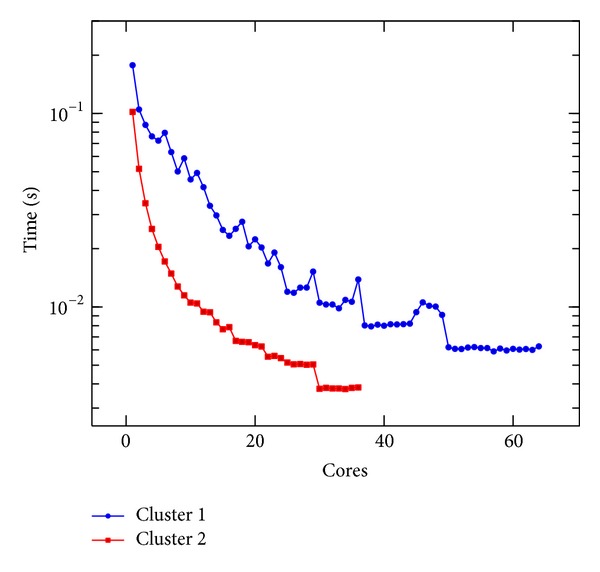
Execution time per iteration of the linear Boltzmann stencil versus number of cores done on Clusters 1 and 2.

**Figure 6:**
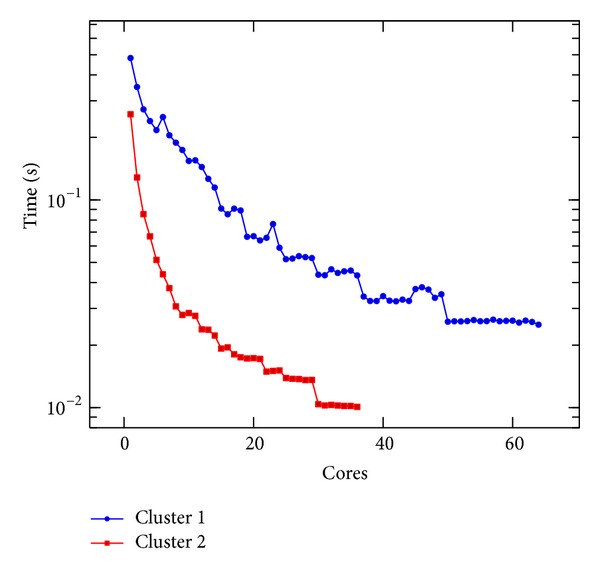
Execution time per iteration of the nonlinear Boltzmann stencil versus number of cores done on Clusters 1 and 2.

**Figure 7:**
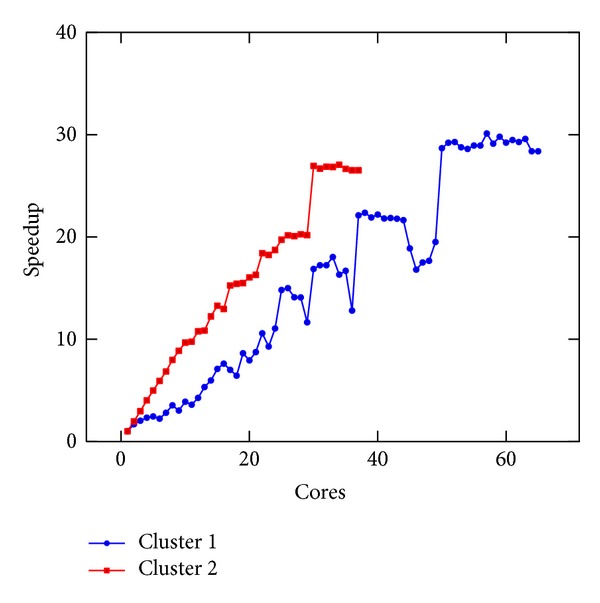
Speedup of the execution time per iteration of the linear Boltzmann stencil versus number of cores done on Clusters 1 and 2.

**Figure 8:**
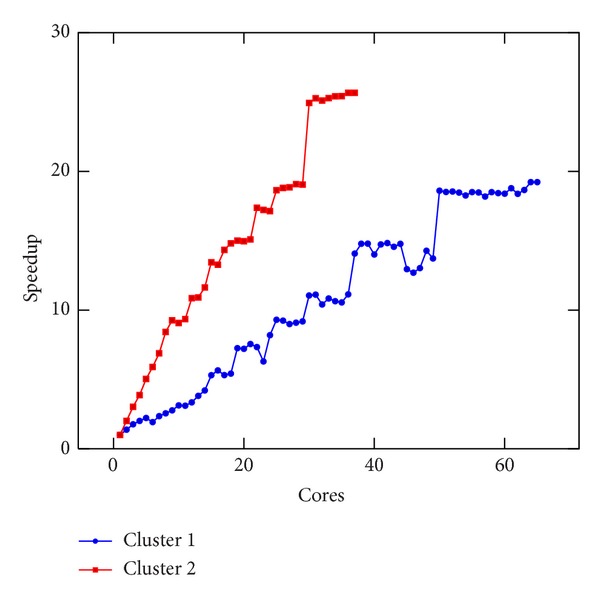
Speedup of the execution time per iteration of the nonlinear Boltzmann stencil versus number of cores done on Clusters 1 and 2.

In Figure 5, we present the execution time of a single iteration of the linear Boltzmann stencil on Clusters 1 and 2. In Figure 6, we present the execution time of a single iteration of the nonlinear Boltzmann stencil on the same resources. An impressive decrease of the execution time was obtained, as it can be seen in Figures 5 and 6. However, the speedup values for both equations, reported in Figures 7 and 8, are not linear. This is to be expected since the problem we are solving is a data intensive problem. Nevertheless, since the speedup for both equations is similar, this is an improvement over the results reported in [15].

### 8.2. CUDA and MPI Performance

**Figure 9:**
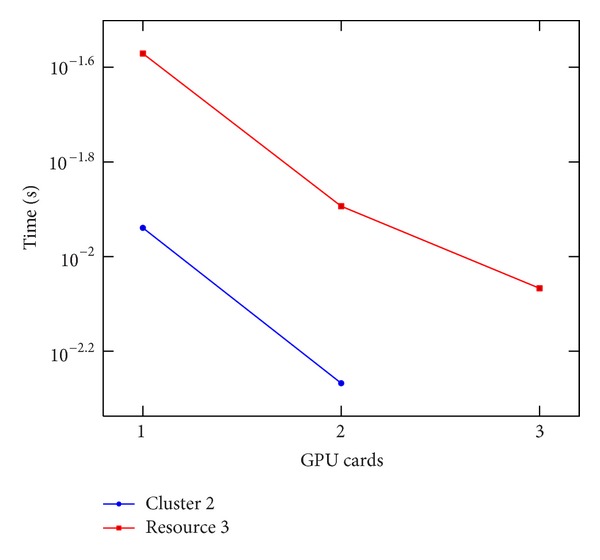
Execution time per iteration of the linear stencil versus number of GPU cards. Done on Cluster 2 and resource 3.

**Figure 10:**
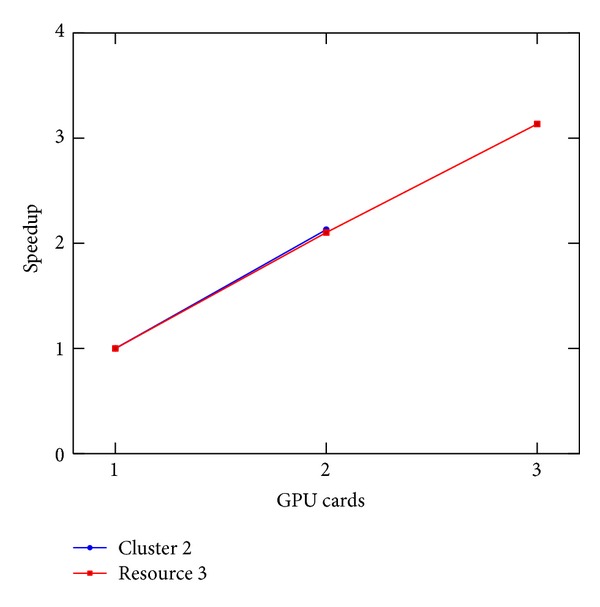
Speedup of the execution time per iteration of the linear stencil versus number of GPU cards. Done on Cluster 2 and resource 3.

Also, presenting the results achieved with the CUDA implementation, to enable a fair comparison of the results obtained on the different clusters, we do not present the time spent in MPI communications and we report the values achieved on the single iteration of the solver. We consider Clusters 2 and 3, respectively, equipped with 2 and 3 GPUs (see Table 2). In Figure 9, the execution time is depicted, while, in Figure 10, the speedup values are depicted. As described in [19], the GPU solver on its own achieves a speedup of about 6.5x when solving the PBE when compared with the serial version. Using 3 cards on one node, an 18.9x speedup is possible. The use of multiple GPUs showed the greatest speedup, achieving a slightly super linear speedup on a single node with three GPU cards. The slightly super linear speedup can be explained by realizing that the number of blocks changes with the change of the subdomain size for each GPU. It may be for that particular grid size and molecule; the number of blocks after the MPI domain subdivision performs better than the original.

### 8.3. MPI and OpenMP Performance

**Figure 11:**
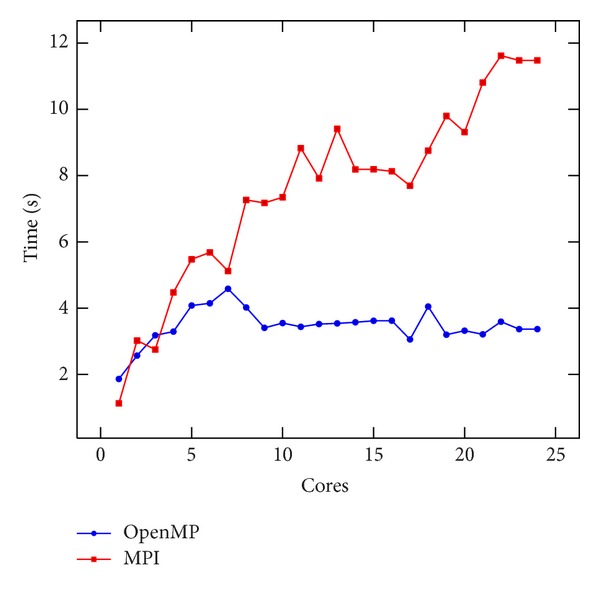
Comparison of the speedup on the stencil between MPI and OpenMP on Cluster 3.

**Figure 12:**
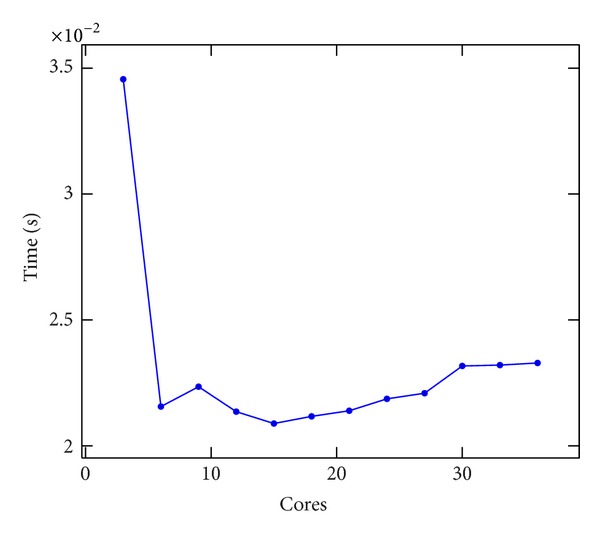
Execution time per iteration of the linear Boltzmann stencil versus number of cores used through OpenMP and MPI working together on Cluster 2.

It is interesting to compare the results obtained using the MPI and OpenMP implementations on the same shared memory node, which can be seen in Figure 11. To test the MPI and OpenMP implementation, we used the three nodes of Cluster 2, spawning three MPI processes and 12 OpenMP threads. Results are depicted in Figure 12. The number of cores was increased equally on all nodes. Neither of the implementations achieved good results. Since the stencil involves a lot of memory access, in a shared memory environment, this could slow down the performance. On the other hand, we have to stress the easiness of the parallelization with OpenMP, definitively much more significant than MPI. The MPI performance paid the effort spent to develop that implementation.

## 9. Conclusions and Future Work

In this paper, we present and compare different parallel implementations of a full PBE solver based on a finite-difference scheme. As for the algorithm itself, we follow the approach of the DelPhi PBE solver, which exploits the checkerboard structure of the finite difference discretization of the Laplace differential operator and adopts a successive overrelaxation scheme to converge to the solution. We parallelize the algorithm using OpenMP, MPI, and CUDA to exploit multicore CPUs, clusters of multicore CPUs, and GPUs. A MPI-CUDA implementation was used to exploit clusters of GPUs. The MPI implementation achieved good speedup values, up to 30 times the serial code using 50 cores. When compared with OpenMP or OpenMP and MPI used together, MPI showed better performance.

Many different points can be investigated as future directions, as the use of nonblocking communication and the features present in CUDA 5 (and onward). The algorithm can also be improved looking for cache optimization, so that the memory access on the Boltzmann stencil is done in a more intelligent way.

## Figures and Tables

**Algorithm 1:**
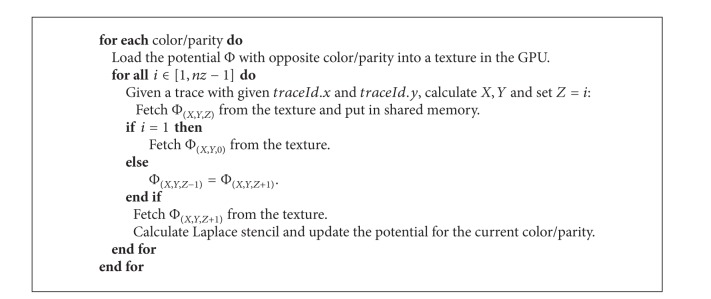
Algorithm for the stencil on a GPU.

**Algorithm 2:**
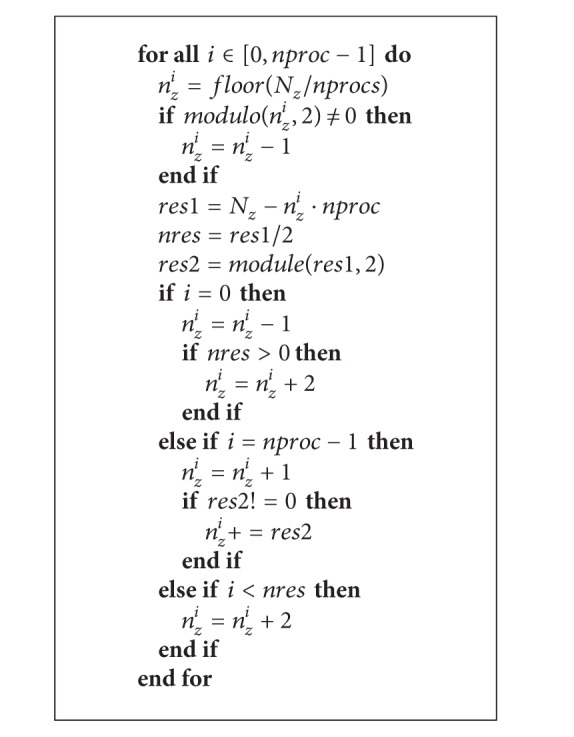
Algorithm for the division of the computation domain.

**Table 1:** Neighbor offsets for even and odd points.

Neighbor	Offset when *p*_*o*_ is even	Offset when *p*_*o*_ is odd
Left (−*X*)	−1	+1

Right (+*X*)	0	0

Back (−*Y*)	$-\frac{\left(n_{x}+1\right)}{2}$	$-\frac{\left(n_{x}-1\right)}{2}$

Front (+*Y*)	$+\frac{\left(n_{x}-1\right)}{2}$	$+\frac{\left(n_{x}+1\right)}{2}$

Bottom (−*Z*)	$-\frac{\left(n_{x}n_{y}+1\right)}{2}$	$-\frac{\left(n_{x}n_{y}-1\right)}{2}$

Top (+*Z*)	$+\frac{\left(n_{x}n_{y}-1\right)}{2}$	$+\frac{\left(n_{x}n_{y}+1\right)}{2}$

**Table 2:** Resources used for testing.

Resource	Nodes	Cores per node	Network	Chip	GPU card	GPUs per node
Cluster 1	8	8	InfiniBand	Quad-Core AMD Opteron Processor 2352	—	—
Cluster 2	2	12	1 Gigabit Ethernet	Intel Xeon E5645	GeForce GTX 580	1
Cluster 3	1	24	—	Intel Xeon X5650	Tesla C2075	3
